# 

**Published:** 1925

**Authors:** 


Vol. XLII. No. 158. WINTER, 1925.

### The Bristol Medico-Chirurgical Journal

PUBLISHED UNDER THE AUSPICES OF

*THE BRISTOL MEDICO-CHIRURGICAL SOCIETY.*

*Editor:* PATRICK WATSON-WILLIAMS, M.D.,

WITH WHOM ARE ASSOCIATED

J. LACY FIRTH, M.S., M.D., CAREY COOMBS, M.D.,
E. W. HEY GROVES, M.S., F.R.C.S., B.Sc.

J. A. NIXON, C.M.G., M.D., *Assistant Editor.*
*Editorial Secretary:* A. L. FLEMMING, M.B., B.Ch.

BRISTOL: J. W. ARROWSMITH LTD., 11 QUAY STREET.

LONDON: J. W. ARROWSMITH (LONDON) LTD., 6 UPPER BEDFORD PLACE, RUSSELL SQUARE.

*Price Three Shillings net.*

